# Feasibility study of volumetric modulated arc therapy with Halcyon™ linac for total body irradiation

**DOI:** 10.1186/s13014-021-01959-3

**Published:** 2021-12-14

**Authors:** Takuya Uehara, Hajime Monzen, Mikoto Tamura, Masahiro Inada, Masakazu Otsuka, Hiroshi Doi, Kenji Matsumoto, Yasumasa Nishimura

**Affiliations:** 1 Department of Radiation Oncology, Faculty of Medicine, Kindai University, Osaka, Japan; 2 Department of Medical Physics, Graduate School of Medical Science, Kindai University, 377-2 Ohno-Higashi, Osaka-Sayama, Osaka 589-8511 Japan

**Keywords:** Total body irradiation, Halcyon™ linac, Volumetric modulated arc therapy, Feasibility study, Radiotherapy

## Abstract

**Background:**

The use of total body irradiation (TBI) with linac-based volumetric modulated arc therapy (VMAT) has been steadily increasing. Helical tomotherapy has been applied in TBI and total marrow irradiation to reduce the dose to critical organs, especially the lungs. However, the methodology of TBI with Halcyon™ linac remains unclear. This study aimed to evaluate whether VMAT with Halcyon™ linac can be clinically used for TBI.

**Methods:**

VMAT planning with Halcyon™ linac was conducted using a whole-body computed tomography data set. The planning target volume (PTV) included the body cropped 3 mm from the source. A dose of 12 Gy in six fractions was prescribed for 50% of the PTV. The organs at risk (OARs) included the lens, lungs, kidneys, and testes.

**Results:**

The PTV D_98%_, D_95%_, D_50%_, and D_2%_ were 8.9 (74.2%), 10.1 (84.2%), 12.6 (105%), and 14.2 Gy (118%), respectively. The homogeneity index was 0.42. For OARs, the D_mean_ of the lungs, kidneys, lens, and testes were 9.6, 8.5, 8.9, and 4.4 Gy, respectively. The V_12Gy_ of the lungs and kidneys were 4.5% and 0%, respectively. The D_max_ of the testes was 5.8 Gy. Contouring took 1–2 h. Dose calculation and optimization was performed for 3–4 h. Quality assurance (QA) took 2–3 h. The treatment duration was 23 min.

**Conclusions:**

A planning study of TBI with Halcyon™ to set up VMAT-TBI, dosimetric evaluation, and pretreatment QA, was established.

**Supplementary Information:**

The online version contains supplementary material available at 10.1186/s13014-021-01959-3.

## Background

Total body irradiation (TBI) is an important conditioning regimen for allogeneic hematopoietic stem cell transplantation. TBI with mega-voltage photon beams is an important treatment modality and is employed for treating several malignant diseases, including multiple myeloma, leukemia, lymphoma, and some solid tumors, and benign diseases, including severe aplastic and Fanconi anemia [1–4].

Most TBI procedures are based on techniques established on linear accelerators used for conventional radiation therapy. Large photon fields are generally achieved by treating a patient at an extended skin-surface distance (SSD) using standard linear accelerators. Equipment guidelines recommend the use of parallel-opposed pairs of high-energy photon beams that range from 4 to 18 MV for TBI [1]. However, conventional TBI has several limitations. Miral et al. reported that patients were required to stand for prolonged periods of time, often more than 40 min [5]. Additionally, conventional TBI has non-conformality of beam application with the inability to individually spare organs at risk (OARs), which results in acute and late toxicity such as pneumonitis or renal dysfunction [6, 7]. Moreover, irradiation of the gonads particularly increased the risk of reduced fertility after the successful completion of cancer treatment in adolescents and young adults (AYAs) [8]. Recovery of gonadal function occurred in only 10%–14% of women and in less than 20% of men [9, 10]. Therefore, fertility preservation in AYA patients treated with TBI should be considered.

The inverse optimization algorithm improved dose homogeneity as compared with conventional forward-planned techniques [11]. A previous study described the dose inhomogeneity of conventional techniques such as extended SSD [12]. In recent studies, helical tomotherapy was applied in TBI and total marrow irradiation (TMI) as an approach to reduce the dose delivered to critical organs, especially the lungs [13, 14]. Furthermore, several studies have reported the feasibility of TBI with linac-based volumetric modulated arc therapy (VMAT) [11, 15–21]. However, the full methodology of TBI with Halcyon™ linac is not well-established.

The present planning study aimed to evaluate whether VMAT with Halcyon™ linac can be clinically used for TBI.

## Methods

### Data sets and contouring

The pre-existing computed tomography (CT) dataset of an adult male patient with a malignant melanoma was used for planning purposes. He was imaged from the vertex of the skull up to the toe, with a total body length of 162 cm.

**Fig. 1:**
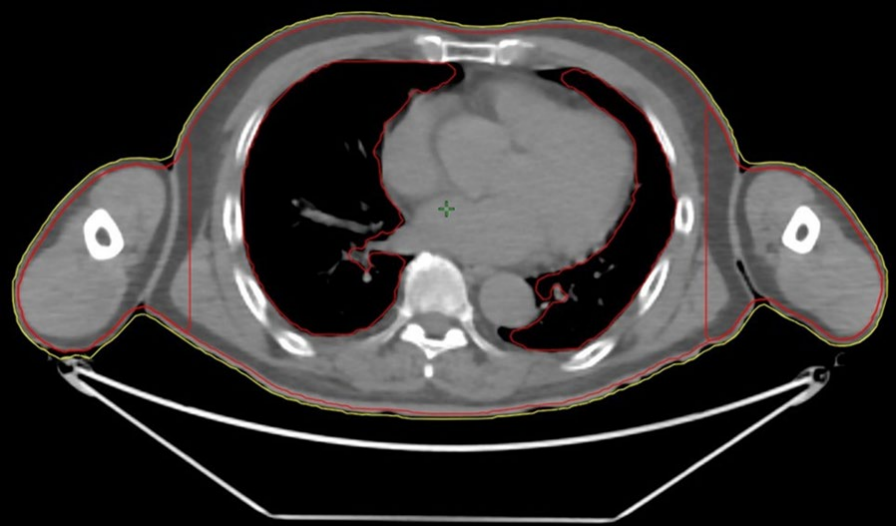
The planning target volume (PTV) includes the entire body (yellow segment) trimmed to 3 mm below the body (red segment). Furthermore, the PTV is divided into two structures at 14 cm from the center in left–right direction as PTV-BODY and PTV-ARM

The planning target volume (PTV) included the entire body trimmed to 3 mm below the body. Furthermore, the PTV was divided into two structures, which are the PTV-BODY and the PTV-ARM (Fig. 1). These structures were 14 cm from the center in the left–right direction. The OARs included the lens, lungs, kidneys, and testes, which were excluded from the PTV.

### Dose prescription and treatment planning

The prescribed dose was 12 Gy in six fractions, which was normalized as 12 Gy to 50% of the PTV-BODY. Our goals for dose-volume histograms (DVHs) are presented in Table 1. These goals were based on previous studies [11, 14, 18, 22].

**Table 1:** The DVH goal for the PTV and OARs

	Parameters	DVH goal
PTV^a^	D_2%_	< 120%
	D_50%_	Around 105%
	D_95%_	> 80%
Lung	D_mean_	< 10.0 Gy
	V_12Gy_	< 2.0%
Kidney	D_mean_	< 10.0 Gy
	V_12Gy_	0%
Lens	D_mean_	< 9 Gy
Testes	D_max_	< 6 Gy
	D_mean_	< 5 Gy

DVH, dose-volume histogram; PTV, planning target volume; OARs, organs at risk; D_2%_, dose to 2% of the volume; D_50%_, dose to 50% of the volume; D_95%_, dose to 95% of the volume; D_mean_, mean dose; V_12Gy_, total volume receiving 12 Gy; D_max_, maximum dose

^a^50% doses were set to 12 Gy

VMAT plans were created using the Eclipse™ treatment planning system (TPS) ver. 15.6 (Varian Medical Systems Inc., Palo Alto, CA). We employed 6 MV flattening filter-free (FFF) photon beams. The VMAT plans were calculated using the Acuros® XB (AXB). We utilized the “base dose plan” function incorporated in the Eclipse™. An O-ring gantry linac with a single 6 MV FFF beam called Halcyon™ (Varian Medical Systems Inc., Palo Alto, CA) was recently introduced. The Halcyon™ linac has unique stacked and staggered dual-layer proximal and distal multileaf collimators (MLC) composed of 10.0-mm-wide leaves that produce an effective 5.0-mm resolution MLC. The maximum field size (28 × 28 cm^2^) at the isocenter is defined by a dual-layer MLC system without physical jaws or light field.

**Fig. 2:**
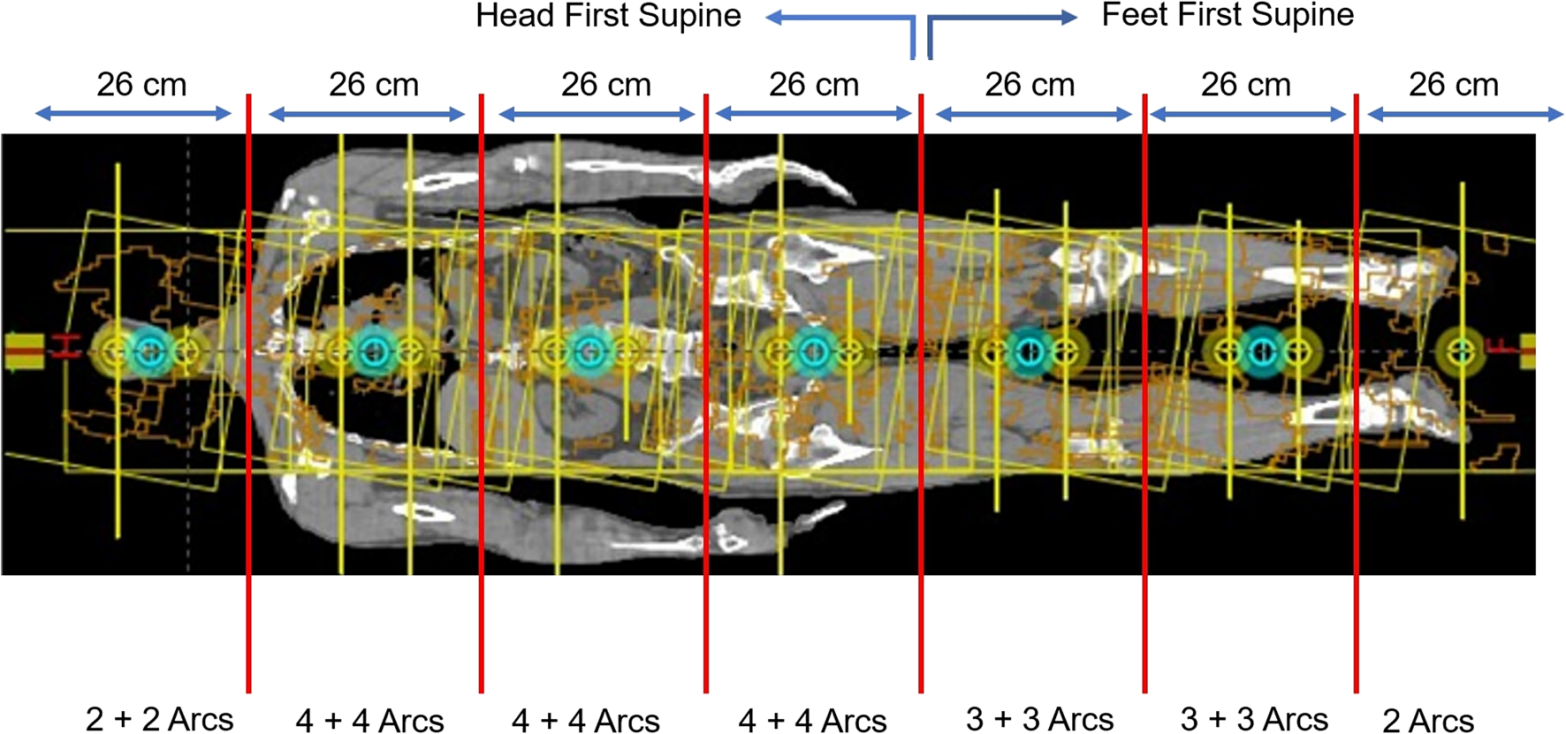
Splitting the planning CT images into a cranial and a caudal part necessitates a dosimetric alignment of these two body parts. The cranial part is created using the head-first position from the vertex to upper thigh, and the caudal part is created using the feet-first position from the toes to lower thigh because the PTV length exceeds the couch travel capability of the Halcyon™ linac. The overall PTV is split into seven segments with a subsequent multi-isocentric planning. There are thirteen isocenters. Each segment is divided at 13 cm from the center in the caudal-cranial direction because of capacity of collimators

Splitting of the CT images into the cranial and caudal parts required a dosimetric alignment of these two body parts. The cranial part was created using the head-first position from the vertex of the skull to the upper thigh, while the caudal part was created using the feet-first position from the toes to the lower thigh because the PTV length exceeded the couch travel capability of the Halcyon™ linac. The overall PTV was split into seven segments with subsequent multi-isocentric planning. Each segment was divided at 13 cm from the center in the cephalad direction because of the capacity of the collimators. There were thirteen isocenters. Figure 2 shows the seven segments of the PTV with thirteen isocenters. Two to four full arcs of VMAT were applied (gantry angle, 181–179° clockwise and 179–181° counterclockwise; collimator angles, 270°, 280°, and 359°) for each isocenter (see Additional file 1: Table S1 for beam arrangement of VMAT-TBI with Halcyon™). The control point spacing was 2° from the angular separation. The beam arrangement was set to meet our goal for PTV coverage of D_95%_ > 80% [23]. The collimator angle was fixed to maximize the field size in each segment. The maximum dose rate was 600 monitor units (MU) per minute. The dose rate was established using the MU calibration geometry setting (isocentric, 90 cm SSD) of the linac and could not be changed by the user.

### DVH analysis and patient-specific quality assurance (QA)

DVH parameters were evaluated in terms of the D_98%_, D_95%_, D_50%_, and D_2%_ of the PTV, where D_98%_, D_95%_, D_50%_, and D_2%_ were the doses received by 98%, 95%, 50%, and 2% of the PTV, respectively. The PTV indicated the overall target volume, including the PTV-BODY and PTV-ARM. In this study, the DVH parameters of the lens, testes, lungs, and kidneys were evaluated, wherein the D_max_ was the maximum dose received by the testes; the D_mean_ was the mean dose received by the lungs, kidneys, lens, and testes; the V_12Gy_ was the total volume of the lungs and kidneys that received 12 Gy; and the V_5Gy_ was the total volume of the lungs that received 5 Gy. Moreover, we evaluated the homogeneity index (HI; defined as [D_2%_ − D_98%_]/D_50%_).

Patient-specific QA was performed to ensure safe delivery of TBI. The difference between the calculated and measured dose distributions was evaluated using the γ pass rate. The ArcCHECK (SunNuclear, Melbourne, FL) and the electric portal imaging device (EPID) of the Halcyon™ linac were employed to evaluate the γ pass rate in each segment. The γ pass rate at each junction between the upper and lower segments, except for the junction between the segments of the head-first and foot-first positions, was evaluated using the ArcCHECK [24]. Furthermore, the absorbed doses at the low-dose gradient point in ArcCHECK in each segment and junction region were measured with a pinpoint ionization chamber (CC01, IBA, Schwarzenbruck, Germany) [25]. The tolerances in terms of dose difference and distance to agreement were 1%/1 mm and 2%/2 mm with a threshold of 10% for the portal dosimetry using the EPID, while they were 2%/2 mm, 3%/2 mm, and 3%/3 mm with a threshold of 10% for the ArcCHECK.

## Results

**Fig. 3:**
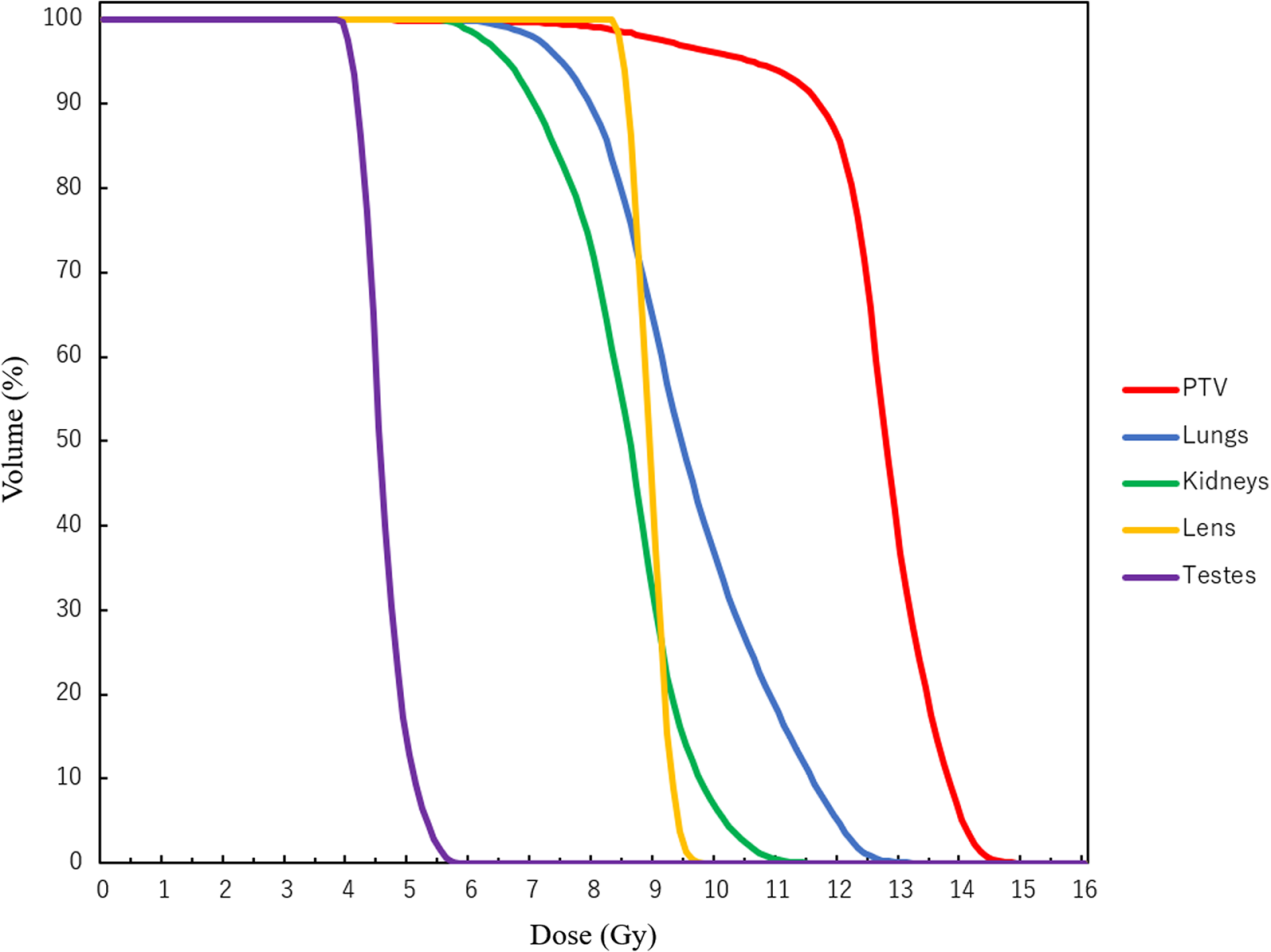
Dose-volume histograms of the planning target volume (PTV), lungs, kidneys, lens, and testes. The D_max_ of the whole body was 15.7 Gy (130%)

**Fig. 4:**
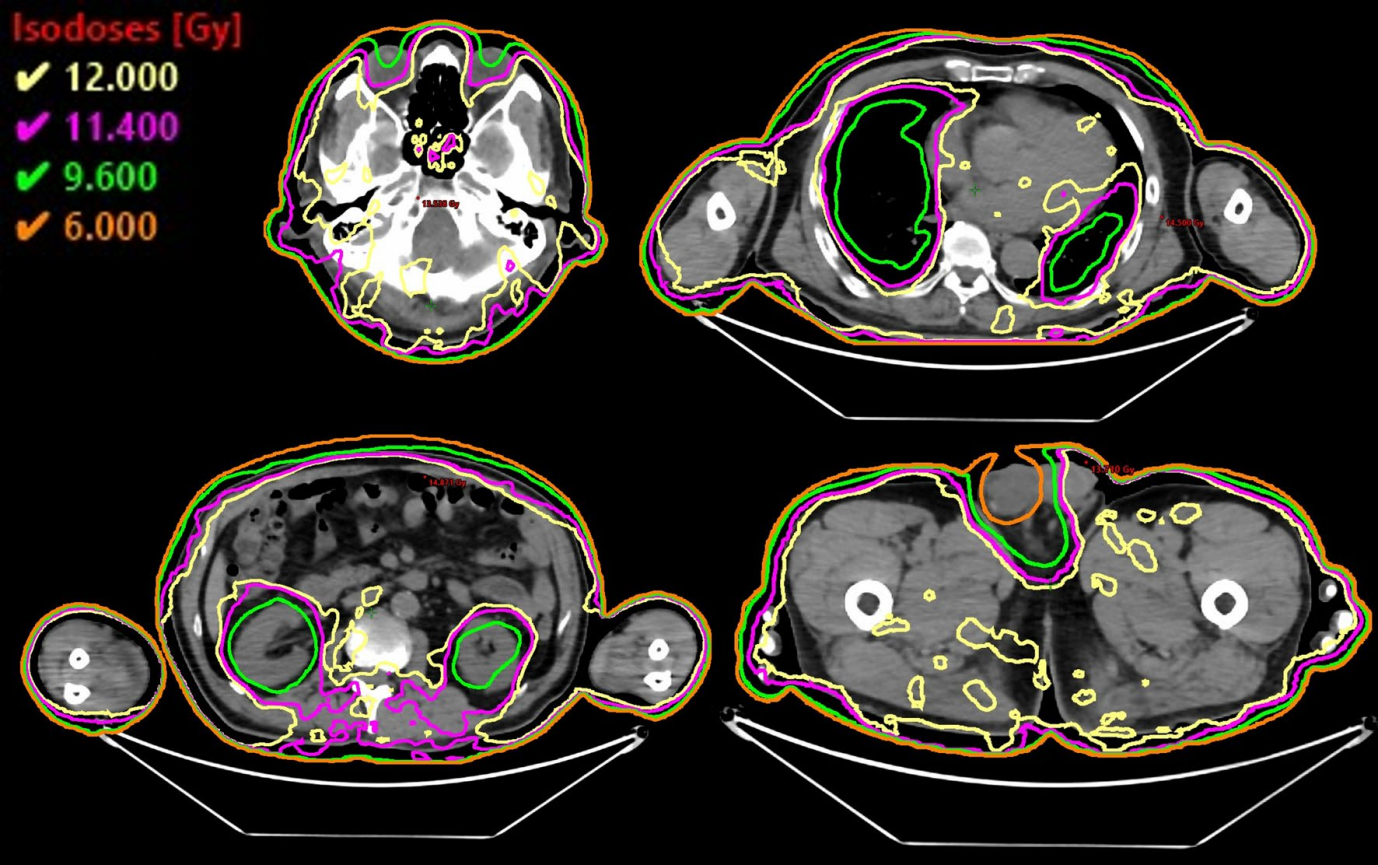
Dose distributions of different computed tomography slices, including the lens, lungs, kidneys, and testes. Yellow line, 12 Gy (100%) iso-dose line; magenta line, 11.4 Gy (80%) iso-dose line; green line, 9.6 Gy (80%) iso-dose line; orange line, 6.0 Gy (50%) iso-dose line

The DVH parameters are summarized in Table 2 and Fig. 3. Figure 4 shows the dose distributions. The D_max_ of the whole body was 15.7 Gy (130%). The PTV D_98%_, D_95%_, D_50%,_ and D_2%_ were 8.9 Gy (74.2%), 10.1 Gy (84.2%), 12.6 Gy (105%), and 14.2 Gy (118%), respectively. The HI was 0.42. In terms of OARs, the D_mean_ of the lungs, kidneys, lens, and testes were 9.6 Gy, 8.5 Gy, 8.9 Gy, and 4.4 Gy, respectively. The D_max_ of the testes was 5.8 Gy. The V_12Gy_ of the lungs and kidneys were 4.5% and 0%, respectively. The V_5Gy_ of the lungs was 100%. The DVH parameters met our goal for the target and almost all OARs, except for the V_12Gy_ of the lungs (Table 2). Additionally, the total MU was 8996.

**Table 2:** DVH parameters for the PTV, OARs, and MU

	Parameters	
PTV	D_98%_	8.9 Gy (74.2%)
	D_95%_	10.1 Gy (84.2%)
	D_50%_	12.6 Gy (105%)
	D_2%_	14.2 Gy (118%)
Lung	D_mean_	9.6 Gy
	V_12Gy_	4.5%
	V_5Gy_	100%
Left lung	D_mean_	9.6 Gy
	V_12Gy_	5.4%
	V_5Gy_	100%
Right lung	D_mean_	9.5 Gy
	V_12Gy_	3.1%
	V_5Gy_	100%
Kidney	D_mean_	8.5 Gy
	V_12Gy_	0%
Left kidney	D_mean_	8.4 Gy
	V_12Gy_	0%
Right kidney	D_mean_	8.5 Gy
	V_12Gy_	0%
Lens	D_mean_	8.9 Gy
Testes	D_max_	5.8 Gy
	D_mean_	4.4 Gy
Total MU		8996
HI		0.42

DVH, dose-volume histogram; PTV, planning target volume; OARs, organs at risk; MU, monitor units; D_98%,_ dose to 98% of the volume; D_95%_, dose to 95% of the volume; D_50%_, dose to 50% of the volume; D_2%_, dose to 2% of the volume; D_mean_, mean dose; V_12Gy_, total volume receiving 12 Gy; V_5Gy_, total volume receiving 5 Gy; D_max_, maximum dose

Table 3 shows the γ pass rates and point dose differences of each segment. The γ pass rates were > 96%, with a criterion of 1%/1 mm using the portal dosimetry with EPID. The γ pass rates were > 90%, with a criterion of 3%/2 mm using the ArcCHECK. All discrepancies in the point doses between the planned and measured doses at each segment were within 3%. Table 4 lists the γ pass rates at each junction. The γ pass rates were also > 90%, with a criterion of 3%/2 mm. In junction 4, which was between the head-first and feet-first positions, the discrepancy in the point dose was 1.91%. All discrepancies in the point doses at each junction were within 5%.

**Table 3:** γ pass rates with EPID and ArcCHECK and point dose differences in each segment

Segment	Seg 1	Seg 2	Seg 3	Seg 4	Seg 5	Seg 6	Seg 7
*EPID*							
1%/1 mm	96.6	96.2	96.3	97.2	97.3	97.3	98.0
2%/2 mm	100	100	100	100	100	100	100
*ArcCHECK*							
2%/2 mm	91.7	89.9	93.6	81.0	81.6	84.4	90.6
3%/2 mm	97.7	97.8	99.4	93.6	94.6	93.8	96.8
3%/3 mm	98.2	98.9	99.7	96.3	97.1	97.0	98.9
Point dose discrepancy (%)	0.60	0.70	2.48	2.16	0.20	1.59	0.43

**Table 4:** γ pass rates with ArcCHECK and point dose differences in each junction

Junction	1		2	3	4	5	6
*ArcCHECK*							
2%/2 mm		84.5	80.5	85.0	–	84.3	91.4
3%/2 mm		94.8	92.9	96.0	–	96.2	98.3
3%/3 mm		97.9	93.3	97.8	–	98.2	98.6
Point dose discrepancy (%)		3.52	4.57	2.78	1.91	4.99	3.37

Treatment planning and QA measurements took longer than typical workflows. Contouring took 1–2 h, dose calculation and optimization took 3–4 h, and QA took 2–3 h. The beam-on time lasted 23 min.

## Discussion

In the present study, a VMAT plan with Halcyon™ linac for TBI was evaluated. This study revealed that this method can be clinically used for TBI. Van et al. and Yao et al. reported that conventional TBI techniques required large treatment fields with lung blocks to irradiate the patient’s entire body, while the patient is in a standing or lying-on-the-side position at an extended SSD (for instance, 5 m), which required costly, large, linear accelerator vaults [26, 27]. In contrast to conventional TBI, the VMAT method with Halcyon™ linac for TBI can be applied in any treatment room that is large enough to fit the Halcyon™ linac. In addition, Gruen et al. described that in helical tomotherapy, the average beam-on time was 34.2 min, with a range of 28.7–39.6 min, for split plans for patients with body lengths over 145 cm [14]. Our beam-on time was 23 min. Thus, the in-room time could be shorter for patients with a body length of 162 cm, even if kilovoltage cone beam CT image guidance was performed for each segment. The standing or lying-on-the-side position at an extended SSD was exhausting for immunocompromised patients undergoing chemotherapy [28]. A shortened period of treatment contributes to patient compliance.

Springer et al. demonstrated that contouring, dose calculation, optimization, and QA took 5–6, 25–30, and 6–8 h, respectively [16]. These parameters were calculated using the RapidArc™ software of the Eclipse™ TPS, version 10.0 (Varian Medical Systems, Palo Alto, CA), in which the body lengths of several patients were over 160 cm [16]. These were much longer than those observed in our study. However, the dose calculation and optimization times in our study were still long because of repeated re-optimization.

In terms of PTV coverage, the D_50%_ and D_mean_ were both 12.6 Gy (105%) in this study. The result was comparable with that of TBI with helical tomotherapy [14] and linac based VMAT [11], respectively. The D_95%_ of the PTV in a previous study [14] was higher (11.7 Gy [97.5%]) than that in this study (10.1 Gy [84.2%]). However, Hirata et al. mentioned that helical tomotherapy increased the absorption dose at the exterior of the irradiation field [29]. Similarly, Mutic et al. demonstrated that the whole-body dose was greater with tomotherapy treatment modalities than with conventional treatments owing to scattered and leakage doses [30]. However, Tamura et al. described that the dose delivery of the Halcyon™ linac was accurate because of the minimal leakage dose and penumbra size of the dual-layer MLC [31].

A further advantage of VMAT with Halcyon™ linac for TBI was the dose reduction in the lens, testes, lungs, and kidneys, as observed in this study. Most centers use lung shielding to maintain the mean lung dose (MLD) of 8–10 Gy, which led to a reduction in the incidence of pneumonitis [32]. The MLD was reduced to 9.6 Gy in this study. The results were comparable to those of other studies for TBI with helical tomotherapy and linac based VMAT [11, 14, 16]. However, in this study, only the V_12Gy_ of the lungs did not meet our goal. Fog et al. reported that their TBI method of step and shoot intensity-modulated radiation therapy with delivering extended SSD achieved a V_12Gy_ of the lungs that was under 2.0% [18]. In our study, the ribs were regarded as the target, such as TMI [33]. The VMAT method ensured full dose coverage of the ribs. Therefore, our results in 4.5% V_12Gy_ of the lungs were considered acceptable. Moreover, the D_mean_ of the kidneys and lens were reduced in this method, and the results were comparable to those of previous studies for TBI with helical tomotherapy and linac based VMAT [11, 14]. These results contributed to reducing the risk of renal dysfunction [34] and cataracts [35]. The most valuable advantage of this method was the reduced dose delivered to the testes. De Felice et al. reported that the threshold dose for permanent sterility in adults was 3–6 Gy [22]. In this study, the D_max_ and D_mean_ of the testes were 5.8 and 4.4 Gy, respectively; therefore, the risk of permanent infertility could be reduced. Hence, the demand for fertility preservation in patients undergoing TBI has increased. However, the frequency of testicular relapse in acute lymphocytic leukemia was very high [36]. Therefore, the decision regarding the VMAT method with testes sparing should be carefully made. The TBI method used in the present study was also used for benign diseases, such as severe aplastic anemia or Fanconi anemia. In contrast, our method of VMAT with Halcyon™ linac could spare any OAR, including the testes.

In the patient-specific QA, the dosimetric accuracy in each segment was within the tolerance limit recommended by AAPM TG218 (γ pass rate of > 90% with a criterion of 3%/2 mm and point dose discrepancy between the planned and measured doses of < 3%) [37, 38]. The γ pass rates in each junction were also > 90% with a criterion of 3%/2 mm using the ArcCHECK. The point dose discrepancies were within 5%, which was within the action limit recommended by AAPM TG218. The reduced accuracy in each junction likely resulted from the detector shift and sagging variation between the upper and lower segments and using the doses in steep gradient areas of the FFF beam for each segment. Thus, this plan was considered acceptable for clinical use.

The present study had several limitations, including small sample size and only one case planning study. However, Chakraborty S et al. described that TBI with VMAT was feasible in one case planning study, the same as in our study [17]. Furthermore, the auto feathering algorithm was not applied for junctions because of the extension of the optimization time (over five hours for each segment). Maddalo et al. described the auto feathering algorithm for the cranio-spinal radiation treatment with the VMAT technique [39]. Therefore, the use of the auto feathering algorithm should be considered for TBI with VMAT. Alternatively, we utilized the “base dose plan” function, which could be achieving optimal plan sum by making up for inadequacies (hot and cold spots) [40].

## Conclusions

A planning study of TBI with Halcyon™ to set up VMAT-TBI, dosimetric evaluation, and pretreatment QA, was established. This method is technically and clinically feasible.

## Supplementary Information

**Additional file 1: Table S1**. Beam arrangement of VMAT-TBI with Halcyon™.

## Data Availability

The datasets used and/or analyzed during the current study are available from the corresponding author on reasonable request.

## Abbreviations

TBI: Total body irradiation
RT: Radiation therapy
SSD: Skin-surface distance
OARs: Organs at risk
AYA: Adolescents and young adults
TMI: Total marrow irradiation
VMAT: Volumetric modulated arc therapy
CT: Computed tomography
PTV: Planning target volume
DVH: Dose-volume histogram
TPS: Treatment planning system
FFF: Flattening filter-free
AXB: Acuros® XB
MU: Monitor units
QA: Quality assurance
D98%: Doses received by 98% of the PTV
D95%: Doses received by 95% of the PTV
D50%: Doses received by 50% of the PTV
D2%: Doses received by 2% of the PTV
Dmax: Maximum dose
Dmean: Mean dose
V12Gy: Total volume receiving 12 Gy
V5Gy: Total volume receiving 5 Gy
HI: Homogeneity index
EPID: Electric portal imaging device
DD: Dose difference
DTA: Distance to agreement
kV: Kilo-voltage
CBCT: Cone beam CT
MLD: Mean lung doses
