# Supplementary material for: Feasibility study of volumetric modulated arc therapy with Halcyon™ linac for total body irradiation
Source: Radiat Oncol. 2021 Dec 14;16:236. doi: 10.1186/s13014-021-01959-3 (PMC8670260; doi:10.1186/s13014-021-01959-3)
Supplement: Supplementary file 1 — Additional file 1: Table S1. Beam arrangement of VMAT-TBI with Halcyon™. [file 13014_2021_1959_MOESM1_ESM.docx]

Table S1. Beam arrangement of VMAT-TBI with Halcyon™

|  | Isocenter number | Gantry rotations (degree) | Collimator angles (degree) | MU |
| --- | --- | --- | --- | --- |
| Segment 1 | 1 | 181 CW 179 | 270 | 227.4 |
|  |  | 179 CCW 181 | 280 | 218.8 |
|  | 2 | 181 CW 179 | 280 | 201.6 |
|  |  | 179 CCW 181 | 270 | 200.7 |
| Segment 2 | 1 | 181 CW 179 | 270 | 270.9 |
|  |  | 179 CCW 181 | 280 | 261.2 |
|  |  | 181 CW 179 | 280 | 262.8 |
|  |  | 179 CCW 181 | 270 | 257.5 |
|  | 2 | 181 CW 179 | 270 | 273.2 |
|  |  | 179 CCW 181 | 280 | 263.5 |
|  |  | 181 CW 179 | 280 | 273.4 |
|  |  | 179 CCW 181 | 270 | 244.4 |
| Segment 3 | 1 | 181 CW 179 | 270 | 286.0 |
|  |  | 179 CCW 181 | 280 | 256.7 |
|  |  | 181 CW 179 | 280 | 272.2 |
|  |  | 179 CCW 181 | 270 | 289.8 |
|  | 2 | 181 CW 179 | 270 | 319.4 |
|  |  | 179 CCW 181 | 280 | 277.8 |
|  |  | 181 CW 179 | 280 | 269.5 |
|  |  | 179 CCW 181 | 270 | 278.2 |
| Segment 4 | 1 | 181 CW 179 | 270 | 241.8 |
|  |  | 179 CCW 181 | 280 | 203.7 |
|  |  | 181 CW 179 | 280 | 227.4 |
|  |  | 179 CCW 181 | 359 | 226.8 |
|  | 2 | 181 CW 179 | 280 | 169.3 |
|  |  | 179 CCW 181 | 270 | 156.2 |
|  |  | 181 CW 179 | 359 | 186.8 |
|  |  | 179 CCW 181 | 270 | 161.3 |
| Segment 5 | 1 | 181 CW 179 | 270 | 141.5 |
|  |  | 179 CCW 181 | 270 | 139.4 |
|  |  | 181 CW 179 | 280 | 150.7 |
|  | 2 | 179 CCW 181 | 270 | 156.4 |
|  |  | 181 CW 179 | 280 | 168.8 |
|  |  | 179 CCW 181 | 280 | 157.7 |
| Segment 6 | 1 | 181 CW 179 | 270 | 113.2 |
|  |  | 179 CCW 181 | 280 | 121.7 |
|  |  | 181 CW 179 | 280 | 117.6 |
|  | 2 | 179 CCW 181 | 270 | 114.8 |
|  |  | 181 CW 179 | 270 | 140.1 |
|  |  | 179 CCW 181 | 280 | 127.8 |
| Segment 7 | 1 | 181 CW 179 | 280 | 280.2 |
|  |  | 179 CCW 181 | 270 | 287.4 |

VMAT, volumetric modulated arc therapy; TBI, total body irradiation; CW, clockwise; CCW, counterclockwise; MU, monitor units
